# Gynecology

**Published:** 1903-09

**Authors:** 


Gynecology.—A Text-Book for Students and a Guide for Practitioners. By William R. Prior, M. D., Professor of Gynecology in the New York Polyclinic Medical School. 380 pages, profusely illustrated. Cloth, $3.50. Sold by subscription only. Published by D. Appleton & Company, 436 Fifth Avenue, New York.

The author has limited himself strictly to gynecology, both in subject matter and illustrations, and so has furnished a short and concise treatise for the use of the busy practitioner and student of medicine. The very rare diseases have been omitted or touched but lightly and in this way room has been reserved for a very full discussion of those commoner diseases of women that the general practitioner is apt to meet. A very convenient arrangement is adhered to throughout the book in that the diseases are described in one part and the operative measures are described in another. This arrangement not only affords systematic reading but also adds much to the value of the work as a ready reference book.

The author shows a decided preference for the vaginal route in operating upon the uterus and appendages, but gives ample space to all methods.

The book is on the whole a very systematic and practical one, and we take pleasure in recommending it to physicians and students of medicine as such.

